# Precision mapping of schistosomiasis and soil-transmitted helminthiasis among school-age children: Targeting interventions in western Kenya

**DOI:** 10.1371/journal.pntd.0013233

**Published:** 2025-07-28

**Authors:** Stella Kepha, Wyckliff Omondi, Maurice R. Odiere, Chitiavi Juma, Martin Muchangi, Dollycate N. Wanja, Chrisistosom Kanyi, Joseph Oloo, Alison Ower, Irene Natalie Chami, Sultani Hadley Matendechero, Florence Wakesho

**Affiliations:** 1 Eastern and Southern Africa Centre of International Parasite Control, Kenya Medical Research Institute, Nairobi, Kenya; 2 Division of Vector Borne and Neglected Tropical Diseases, Ministry of Health, Nairobi, Kenya; 3 Centre for Global Health Research, Kenya Medical Research Institute, Kisumu, Kenya; 4 Amref Health Africa, Nairobi, Kenya; 5 Safe Water and AIDS Project, Kisumu, Kenya; 6 The END Fund, Nairobi, Kenya; 7 Ministry of Health, National Public Health Institute, Nairobi, Kenya; Stony Brook University, UNITED STATES OF AMERICA

## Abstract

**Background:**

Mapping of schistosomiasis (SCH) and soil-transmitted helminthiasis (STH) is a critical step in understanding where at-risk populations live in order to effectively plan and target available resources and to achieve maximum impact on disease burden. A precision mapping protocol was developed and implemented in Kakamega, Bungoma, Trans Nzoia and Vihiga Counties in western Kenya by applying the current World Health Organization (WHO) mapping guideline at a lower administrative level (Ward).

**Methods:**

Cross-sectional surveys were conducted among school-age children (SAC) in 5 primary schools purposefully selected in each mapping unit (Ward). In each school, stool and urine samples were collected from 60 randomly selected children (ages 8–14 years). The prevalence and intensity of infection of *Schistosoma mansoni* and STH were determined using the Kato-Katz technique and urine filtration for *S. haematobium*. Water Sanitation and Hygiene (WASH) status were also recorded.

**Results:**

Of the 46,464 children sampled, 3.2% (95% CI: 3.0-3.3) were infected with at least one *Schistosoma* species, with *S. mansoni* being the most predominant at 3.2% (95% CI: 2.9 - 3.3). 7.6% (95% CI: 7.3 – 7.8) of children were infected with at least one STH species, with *A. lumbricoides* being the most common (6.5%), and hookworm the least common (0.7%). The prevalence of *S. mansoni* was highest in Bungoma County (4.5%) and lowest in Trans Nzoia county (0.5%); STH prevalence was highest in Vihiga County (10.7%) and lowest in Trans Nzoia County (4.8%). SCH and STH infections were mainly of light intensity (2.2% and 5.6%, respectively). Based on sub-County-level data and prevalence threshold of ≥2% for MDA, 49 and 144 Wards required treatment for SCH and STH, respectively, whereas based on the Ward-level data, only 40 and 138 Wards required treatment for SCH and STH, respectively.

**Conclusions:**

Use of Ward relative to sub-county prevalence revealed considerable spatial heterogeneity for SCH and STH and resulted in 14.5% and 0.8% reduction in the number of people treated for SCH and STH, respectively, underscoring the critical role of precision mapping in improved targeting of interventions.

## Author summary

Mapping schistosomiasis (SCH) and soil-transmitted helminthiasis (STH) is essential to identify at-risk populations and implement targeted interventions effectively. Despite global progress, existing SCH mapping approaches still struggle to account for SCH’s clustered distribution within implementation units. Since 2012, Kenya’s National School-Based Deworming Programme has focused on treating school-age children (SAC) based on a sub-national baseline survey and spatial modeling from that year. Under the Breaking Transmission Strategy (BTS), Kenya’s Ministry of Health adopted a WHO-guided Ward-level precision mapping protocol to support the elimination of neglected diseases like SCH and STH. This approach enabled a more accurate identification of at-risk populations, revealing variations in prevalence and ultimately reducing treatment needs for SCH and STH through more efficient community-based mass drug administration in western Kenya.

## Introduction

Schistosomiasis (SCH) and soil-transmitted helminthiasis (STH) continue to pose significant public health challenges, with school-aged children bearing the greatest burden, due to their frequent contact with parasite-infested environments [1]. Schistosomiasis, caused by parasitic worms of the *Schistosoma* genus, remains widespread, with approximately 90% of cases concentrated in sub-Saharan Africa [2]. In Kenya, both *Schistosoma mansoni* (intestinal schistosomiasis) and *Schistosoma haematobium* (urogenital schistosomiasis) are endemic, with national estimates indicating approximately 9 million infections [3]. STH infections also remain a pressing concern, with the three most common STH species *Ascaris lumbricoides, Trichuris trichiura*, and hookworms (*Ancylostoma duodenale* and *Necator americanus*) affecting over 1.5 billion people worldwide [1]. *A. lumbricoides* is the most widespread, accounting for approximately 820 million infections globally [4]. In Kenya, western and coastal counties are the most affected, despite long-standing national deworming programs and preventive chemotherapy (PC), these infections continue to exert considerable health and socio-economic tolls, especially among vulnerable children [5]. Both SCH and STH have been targeted for elimination as public health problems (EPHP) both within the World Health Organization (WHO)’s Roadmap for Neglected Tropical Diseases (NTDs) 2021–2030 [1]. Global and national efforts to achieve EPHP have largely relied on preventive chemotherapy delivered via mass drug administration (MDA) campaigns [1,6,7]. These campaigns typically target school-aged children and treatment eligibility are determined based on prevalence estimates derived from a limited number of schools within each district (sub-county in the context of Kenya). However, the focal nature of SCH where transmission intensity can vary markedly even within small geographic areas makes such sampling prone to misclassification. This can result in overtreatment in low-risk zones and critical under-treatment in high-burden localities that remain undetected [7–13]. To better align control intervention efforts with transmission realities on the ground, there has been a growing call for more granular approaches to disease mapping, the World Health Organization’s (WHO) 2021–2030 Roadmap for Neglected Tropical Diseases emphasizes the importance of precision mapping as a critical strategy to improve the efficiency and impact of programmatic interventions [1]. This refined mapping approach enables national programs to more accurately delineate at-risk populations, optimize the allocation of preventive chemotherapy, and accelerate progress toward elimination goals

In response to the limitations of the current conventional mapping design for SCH, the Kenya Ministry of Health (MoH)’s NTD programme conducted precision mapping for SCH n four counties in western Kenya. ‘*Precision mapping*’. This approach involves purposive sampling of a larger number of schools or communities within lower levels administrative units (Wards) below each IU (previously sub-county), thereby capturing the fine-scale heterogeneity in transmission. so as to address the variability in prevalence attributable to the focal nature of SCH. The WHO mapping guideline also recommends mapping of SCH and STH together whenever both diseases are co-endemic. In this regard, STH were included in the precision mapping alongside SCH. We detail here the methodologies, results and treatment decisions made following the precision mapping exercise in the four counties of Kakamega, Bungoma, Vihiga and Trans Nzoia in western Kenya. Findings are considered within the context of both Kenya’s BTS as well as the current WHO guideline on control and elimination of human schistosomiasis.

## Methods

### Ethical considerations

Ethical approval for the survey was granted by the Research Ethics Committee of the University of Eastern Africa Baraton (UEAB/REC/01/08/2021). Additional permissions were provided by the appropriate county-level health and education authorities, who were briefed appropriately about the survey. Parents and participants were provided with information sheets detailing the purpose of the study and what to expect. Written informed consent by parents or guardians and verbal assent by participating children were obtained prior to enrolment in the study.

### Study sites and population

**Fig 1:**
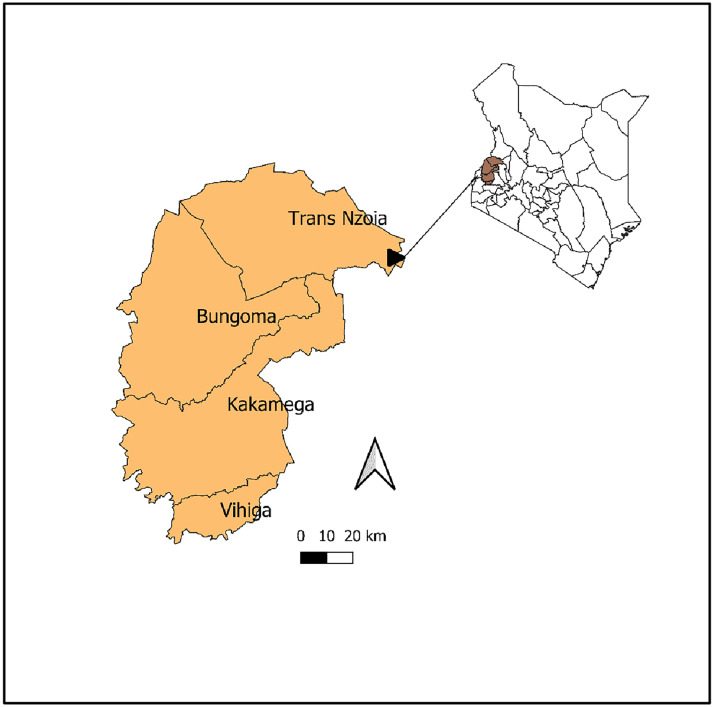
Four counties in western Kenya where the study was conducted https://www.gadm.org/download_country.html.

The study was conducted in the 4 counties of Kakamega, Bungoma, Vihiga and Trans Nzoia in western Kenya (**Fig 1**) in September 2021. The region covers approximately 12,891Km^2^.

The region has a tropical to cool and temperate climate with temperatures ranging from 10^0^C to 32^0^C. It experiences two rainy seasons, the long rains of March to July and short rains August to October. The average annual rainfall is between 1,280–2,214 mm (Kakamega), 400–1,800mm (Bungoma), 1900mm (Vihiga) and 1000–1200mm (Trans Nzoia). The major water bodies include rivers such as Yala, Isiukhu, Nzoia, Kuywa, and Saiwa swamp and several dams.

The 4 counties have a combined population of approximately 5,118,503 [14]. The main ethnic communities are Luhya, Teso and Sabaot. Communities in the region practice a mixture of both subsistence and cash crop farming, with maize and sugar cane being the preferred large-scale cash crop. Other economic activities include cottage Industries, tea farming, horticulture, livestock farming, wholesale and retail trade, quarrying and mining. This region is known to be endemic for *S*. *mansoni* and STH [10] but with a paucity of data on *S. haematobium*. Whereas there was some existing data on SCH and STH for these counties [10,15–18], the data is from limited sources with gaps in methodology and in addressing focality of SCH that would hamper the effective implementation of the BTS. The relatively low prevalence of SCH and STH infections in the 4 counties informed their selection for the roll-out of BTS, interruption of transmission is deemed feasible.

### Survey methodology

The survey applied the conventional WHO mapping guideline but was cascaded to the lowest administrative level (Ward). A Ward is an administrative unit just below the sub-County and is the smallest electoral division in the country and closest the citizens can get government services. For each administrative level (Ward), based on the current WHO recommended mapping design for schistosomiasis, 5 primary schools were included (the Ward was the mapping unit). For each mapping unit, one overall prevalence value was calculated to enable classification of the Ward into non-endemic, low, moderate or high-risk area [19], and thereafter one treatment strategy was applied based on this classification. A similar strategy was previously implemented in Kenya’s coastal region, serving as a foundational effort toward improving spatial resolution in disease surveillance. The current survey represents a continuation of these efforts by the Kenya Ministry of Health (MoH) to complete the national schistosomiasis endemicity map through the generation of fine-scale, ward-level data, thereby supporting targeted intervention planning and aligning with both national and global NTD elimination goal [20].

### Site selection and sample size for sampling in SAC

All the 155 Wards in the 4 counties were included in this survey. In each Ward, 5 schools were selected for a total of 774 schools included in the survey (1 Ward had a maximum of 4 schools). Selection of schools was purposeful and was guided by several considerations including previous knowledge of the areas where transmission is known, suspected or more likely near water bodies: lakes, streams, dams and irrigation areas. Identification of water bodies was aided by suitability maps based on the available spatial data. Previous knowledge was collected through desktop reviews, data from ESPEN, data from the National school-based deworming program (NSBDP) which was obtained from the Children’s Investment Fund Foundation (CIFF) reports and local knowledge of disease transmission from Division of Vector Borne and Neglected Tropical Diseases (DVBNTD) laboratories and data from peripheral health facilities. Selection of villages considered the geographical distribution of schools in order to be representative.

### Recruitment and sample collection

The inclusion criteria for participants were (a) ages 8–17 years with parental/guardian consent, (b) assent from children (those above 13 years provided written assent) to participate in the study, (c) resident in the study area/village for the last six months, and (d) no reported receipt of deworming medication in the last 6 months.

Advocacy and sensitization activities were conducted from county through to village level. The cascade was as follows; the national team conducted the county advocacy, the county team conducted sub-county advocacy, the sub-county teams conducted the Ward advocacy from where the schools were selected.

In each school, 60 children were targeted for enrolment in the study, for approximately 300 children per Ward. Children were randomly sampled in each school. On the day of the survey the field officers arrived at the school and explained to the head teacher and children the purpose of the study, requirements for sample collection, and ensured that appropriate assent/consent was provided by the selected children and parents or guardians prior to their participation. Once the consent and assent were obtained, children were provided with instructions and materials for collection and return of their stool and urine sample. Each participant was assigned a unique study identification number, which was used to track all collected samples. The fresh stool and urine samples collected were transported in cold condition (cooler box with ice packs) to the nearest MoH laboratory where they were analysed for presence or absence of helminth infections by Kato-Katz and urine filtration techniques, respectively.

Demographic information of the participants including age and sex were collected using the Kobo collect digital platform. Data on WASH variables was collected from all individuals using a standard WASH questionnaire.

### Parasitological assessments

All stool and urine samples were transferred to the laboratory within two hours of collection for processing on the same day. Stool samples were prepared using the Kato-Katz thick smear method [21]. Duplicate slides (41.7mg template) and examined microscopically for the presence and number of *S*. *mansoni* and STH eggs by two independent technicians. For quality assurance, 10% of all the positives and negative thick smears were re-examined by a third laboratory technician blinded of the results of the other two technicians. Infection intensity was calculated and expressed as the number of eggs per gram of stool (EPG). Urine filtration diagnostic technique was used to detect *S. haematobium* eggs in 10ml urine [21], and infection intensity determined as number of eggs counted per 10ml urine.

### Data management and analysis

The parasitological and WASH questionnaire data were entered in Kobo Collect (Kobo Inc., Cambridge, MA, USA) and directly uploaded to the cloud for later analysis. To minimize opportunity for user error, entries were selected from a drop-down list where possible. Data management and analyses were performed using STATA version 15.0 (STATA Corporation, College Station, TX, USA). Participants with complete demographic and parasitological data were included in the final analysis. The final sample of children included in the analysis was limited to those with complete data for sex and age, with the latter between years 8 and 14 inclusive, as well as at least one valid stool or urine sample. Any STH in individuals with a valid stool sample was defined as having at least one positive infection of any STH species. In the case of SCH, where stool and urine sample within children were not always available/valid, classification of “Any SCH” was limited to children with both valid stool and urine samples. The overall prevalence of each SCH and STH species and STH combined was calculated at the Ward, sub-county and county levels and the 95% confidence intervals (CIs) obtained using binomial regression analysis taking into account clustering by school, Ward and subcounty respectively. Arithmetic mean egg intensity for infected individuals was calculated based on the average of the egg counts of the duplicate slides and presented at the County and Ward levels. The intensity of helminth infection (egg density) was further categorized into light, moderate and heavy infections according to WHO-proposed thresholds for the classification of individuals with helminth infections [22]. The effect of sex and age class on whether a child was infected was analysed using binary logistic regression. The prevalence of each intensity infection class together with 95% CIs were obtained using negative binomial regression analysis taking into account clustering by school, Ward and subcounty respectively. The prevalence of SCH and STH was compared among counties using a chi-square test.

The updated WHO guidelines identify a prevalence threshold of 10% for annual preventive chemotherapy for schistosomiasis, whereas Kenya’s BTS threshold for initiating MDA is set at 2% [6,23]. Appreciating that not all areas in the country are at the same stage of control, coupled with the fact that SCH is epidemiologically distinct throughout its geographical distribution [9], the BTS threshold of 2% is considered critical in areas where SCH prevalence is already sufficiently low, with great feasibility to interrupt transmission (i.e., BTS is not rolled out in a “one size- fits-all” approach). These categories were used to classify Wards in the analysis, and presented in such a way that allows the reader to interpret the findings in the context of the varying guidelines and strategies as follows: zero prevalence (0%), low and no MDA (*>*0% and *<*2%), low and MDA (Kenya BTS) (≥2% and *<*10%); WHO low (*>*0 and *<*10%), WHO moderate (≥10 and *<*50%) or high prevalence (≥50% prevalence). Similar to SCH, Kenya’s BTS threshold for MDA for STH is ≥ 2%. Therefore, STH was classified into low and no MDA (*>*0% and *<*2%), low prevalence and MDA (Kenya BTS) (≥2% and *<*20%), WHO low risk (≥20% and *<*50%) and high risk (≥50%). The treatment strategies, cut-offs and recommendations according to Kenya’s BTS and WHO are illustrated elsewhere [20]. To establish Ward-specific risk maps, the geographic coordinates of each school sampled were linked to survey data using 3.10 QGIS software.

## Results

### Sample population and characteristics

**Fig 2:**
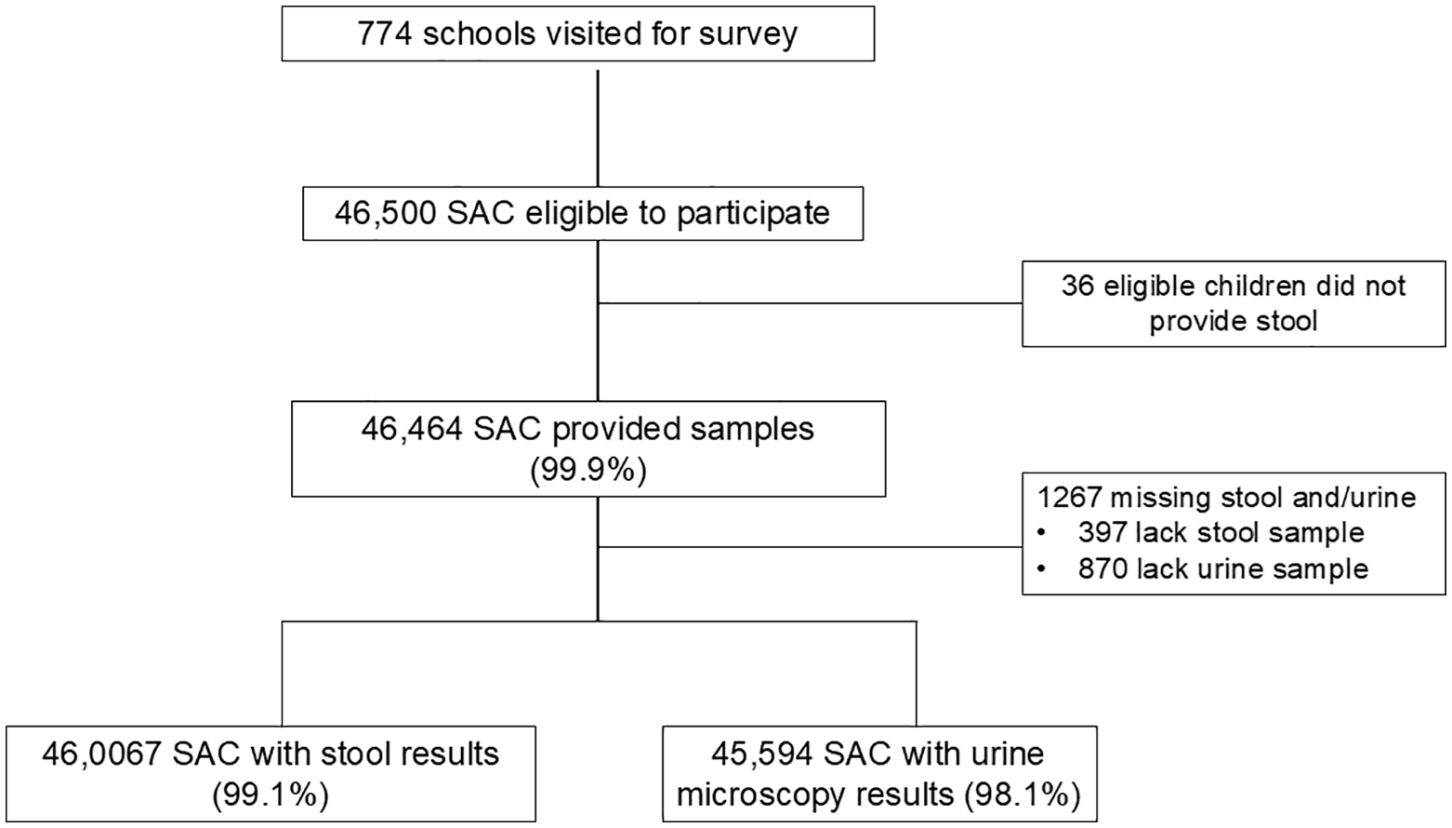
Data flow diagram showing recruitment rates and loss to follow-up at various stages of sampling.

The survey included a total of 46,500 children. Not all children who participated had complete data for age and/or provided stool and/or urine samples (**Fig 2**).

Overall, 50.5% (n = 22,383) of SAC were females. The mean age was 11.4 years, age range 5–17 years.

#### Prevalence of helminth infections.

In total, 1,471 individuals out of 46,464 (3.2%) who provided either a stool or urine sample were infected with any schistosome species (**Table 1**). *S. mansoni* was the most prevalent schistosome species. A total of 1,437 individuals out of 46,067 (3.2%) who provided a stool sample were infected with *S. mansoni*. The prevalence of light, moderate and heavy *S. mansoni* infections was 2.1% (n = 982), 0.8% (n = 371) and 0.2% (n = 84), respectively (**Table 1**). A total of 35 individuals out of 45,594 (0.08%) were infected with *S. haematobium.* The prevalence of light and heavy *S. haematobium* infections was 0.07% (n = 33) and 0.004% (n = 2), respectively.

**Table 1:** Overall prevalence and intensities of helminth infections among SAC from the granular mapping survey.

Helminth species	Number tested (n)*	Number positive	Prevalence, % (95% CI)	Proportions of infection intensities				Mean EPG x̄, (95% CI)	Mean eggs/10mL urine
				Light n, (%)	Moderate n, (%)	Heavy n, (%)	Moderate & Heavy n, (%)		
Any SCH	46464	1471	3.2 (3.0-3.3)	1014 (2.2)	371 (0.8)	86 (0.2)	N/A	N/A	N/A
*S. mansoni*	46067	1437	3.2 (2.9-3.3)	982 (2.1)	371 (0.8)	84 (0.2)	N/A	125.4 (114.6-136.2)	N/A
*S. haematobium*	45594	35	0.08 (0.06-0.11)	33 (0.07)	N/A	2 (0.004)	N/A	N/A	11.3 (5.2-17.4)
Any STH	46067	3489	7.6 (7.3-7.8)	2592 (5.6)	873 (1.9)	24 (0.1)	897 (2.0)	N/A	
*A. lumbricoides*	46067	2989	6.5 (6.3-6.7)	2102 (4.6)	866 (1.9)	22 (0.1)	888 (1.9)	5560.7 (5205.3-5916.0)	N/A
Hookworm	46067	305	0.7 (0.6-0.8)	305 (0.7)	0	0	0	136.1 (110.5-161.7)	N/A
*T. trichiura*	46067	409	0.9 (0.8-1.0)	397 (0.9)	10 (0.02)	2 (0.004)	12 (0.02)	194.7 (107.2-282.2)	N/A

* Sample sizes represent all those who provided stool or urine samples regardless of whether they had data for sex or age.

A total of 3,489 individuals out of 46,067 (7.6%) who provided a stool sample were infected with any STH infection (**Table 1**). The prevalence of *A. lumbricoides,* hookworm and *T. trichiura* was 6.5%, 0.7% and 0.9%, respectively. *A. lumbricoides* was the most prevalent STH while hookworm was the least prevalent (**Table 1**).

#### Effect of sex and age on helminth infections.

Sex had no effect on infection prevalence for any STH or single STH species infections. On the other hand, females had a lower risk of being infected with any SCH (OR = 0.7; 95% CI: 0.6-0.8; P < 0.001) and *S. mansoni* (OR = 0.7; 95% CI: 0.6-0.8; P < 0.001), but no difference was noted by sex for *S. haematobium*. There was no difference for any STH or single STH species infection by age, except for hookworm where the prevalence was over 2-fold higher among children aged ≥15 to <18 years compared to those aged ≥5 to <10 years (OR = 2.2; 95% CI: 1.1-4.4; P = 0.024). With regards to schistosomiasis, there was a trend toward increase in prevalence of infections with age for any SCH and for *S. mansoni*, but not for *S. haematobium.* Children aged ≥10 to <15 years and those aged ≥15 to <18 years were 1.6 and 1.9 times more likely, respectively, to be infected with any SCH compared to those aged ≥5 to <10 years. Children aged ≥10 to <15 years and those aged ≥15 to <18 years were 1.7 and 2.0 times more likely, respectively, to be infected with *S. mansoni* compared to those aged ≥5 to <10 years.

### Prevalence of Schistosomiasis across counties, sub-counties and wards

**Fig 3:**
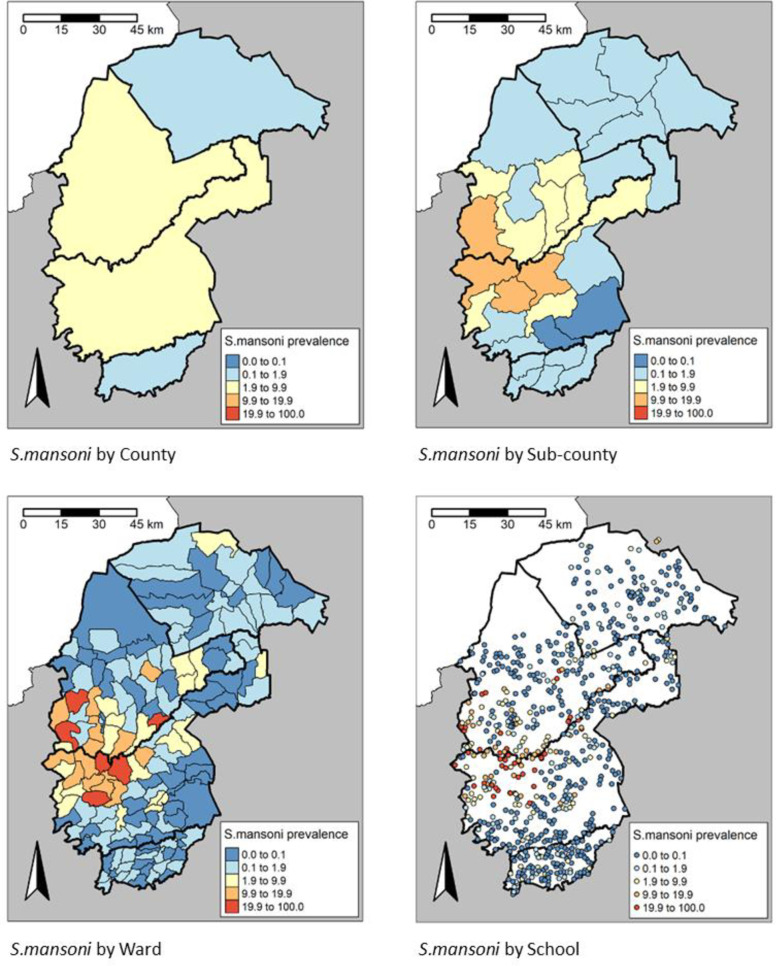
Prevalence distribution of *S. mansoni* by County, sub-County, Ward and site level, https://www.gadm.org/download_country.html.

**Fig 4:**
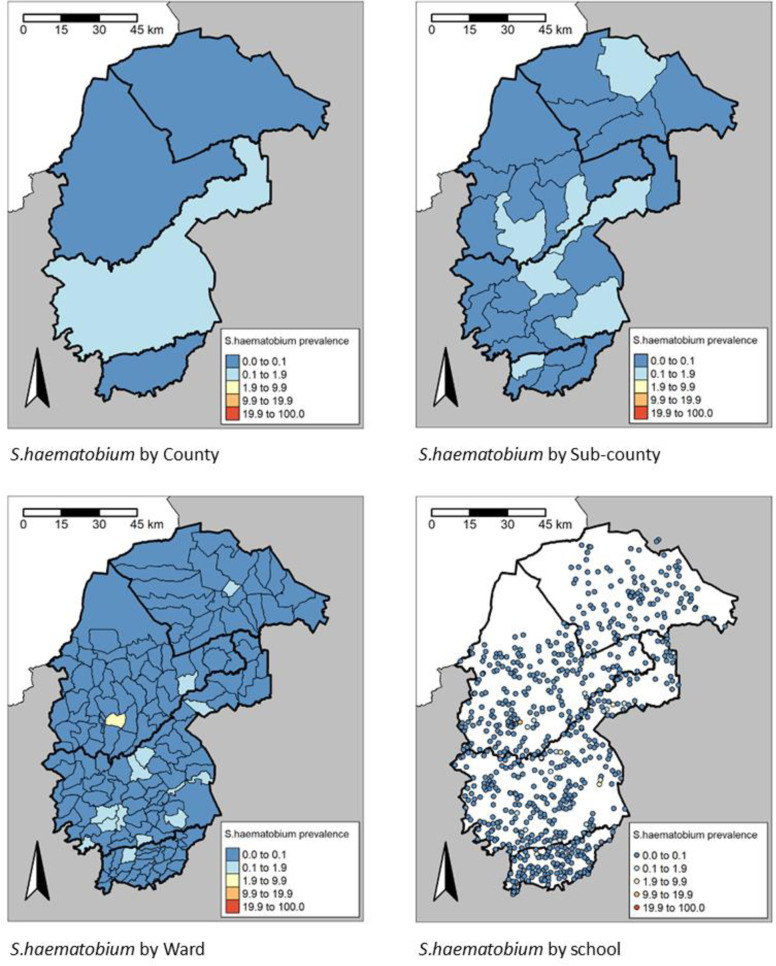
Prevalence distribution of *S. haematobium* by County, sub-County, Ward and site level. https://www.gadm.org/download_country.html.

The observed survey prevalence for any schistosomiasis varied from 0.5% (95% CI: 0.4-0.7) in Trans Nzoia County to 4.6% (95% CI: 4.2-5.0) in Bungoma County (**Table 2**), and from 0% in Mt. Elgon sub-County in Bungoma county to 17.6% (95% CI: 15.7-19.6) in Matungu sub-County in Kakamega county (**Table 3**), although these County and sub-County aggregates hide considerable spatial heterogeneity, with individual SCH species prevalence seen to vary markedly between Wards and Sites (**Figs 3** and **4**). *S. mansoni* was focally distributed in the study area but most prevalent in Bungoma County with an overall prevalence of 4.5% (4.2-4.9) (**Table 2**). In the 262 schools where *S. mansoni* infected children were found, the proportion positive ranged from 1.6% to 61.7%. The distribution of *S. mansoni* showed a strong spatial trend, localised primarily to the western side along the border between Bungoma and Kakamega counties. In the 30 schools where *S. haematobium* infected children were found, the proportion positive ranged from 1.7% to 10%.

**Table 2:** Prevalence and intensity of schistosomiasis and soil-transmitted helminths among SAC in the 4 counties of the western region.

County	Any SCH	*S. mansoni*	*S. haematobium*	Any STH	*A. lumbricoides*	Hookworm	*T. trichiura*
**Bungoma**							
Number surveyed	13491	13450	13350	13450	13450	13450	13450
Number positive	619	610	9	927	808	98	54
Prevalence, % (95% CI)	4.6 (4.2–5.0)	4.5 (4.2–4.9)	0.07 (0.04–0.13)	6.9 (6.5–7.3)	6.0 (5.6–6.4)	0.7 (0.6–0.9)	0.4 (0.3–0.5)
% light		3.0	0.07	5.1	4.2	0.7	0.4
% moderate		1.3	N/A	1.8	1.8	0.0	0.03
% heavy		0.3	0.0	0.0	0.0	0.0	0.0
% moderate & heavy		N/A	N/A	1.8	1.8	0.0	0.03
Av. Intensity (epg):		133.0	8.0 eggs/10 mL		5148.4	107.6	368.4
**Kakamega**							
Number surveyed	17928	17657	17518	17657	17657	17657	17657
Number positive	768	747	22	1401	1164	160	177
Prevalence, % (95% CI)	4.3 (4.0–4.6)	4.2 (3.9–4.5)	0.13 (0.08–0.19)	7.9 (7.5–8.3)	6.6 (6.2–7.0)	0.9 (0.8–1.1)	1.0 (0.9–1.2)
% light		3.0	11.4	6.4	4.9	0.9	1.0
% moderate		1.0	N/A	1.6	1.6	0.0	0.02
% heavy		0.2	0.01	0.1	0.1	0.0	0.01
% moderate & heavy		N/A	N/A	1.7	1.7	0.0	0.03
Av. Intensity (epg):		119.9	13.3 eggs/10 mL		4876.2	157.1	237.2
**Vihiga**							
Number surveyed	7489	7411	7323	7411	7411	7411	7411
Number positive	44	41	3	796	698	27	145
Prevalence, % (95% CI)	0.6 (0.4–0.8)	0.6 (0.4–0.8)	0.04 (0.00–0.12)	10.7 (10.1–11.5)	9.4 (8.8–10.1)	0.4 (0.2–0.5)	2.0 (1.7–2.3)
% light		0.4	0.04	7.3	6.1	0.4	1.9
% moderate		0.1	N/A	3.3	3.3	0.0	0.04
% heavy		0.04	0.0	0.1	0.1	0.0	0.0
% moderate & heavy		N/A	N/A	3.4	3.4	0.0	0.04
Av. Intensity (epg):		151.0	9.0 eggs/10 mL		6973.7	164.9	97.7
**Trans Nzoia**							
Number surveyed	7556	7549	7403	7549	7549	7549	7549
Number positive	40	39	1	365	319	20	33
Prevalence, % (95% CI)	0.5 (0.4–0.7)	0.5 (0.4–0.7)	0.01 (0.00–0.09)	4.8 (4.4–5.3)	4.2 (3.8–4.7)	0.3 (0.2–0.4)	0.4 (0.3–0.6)
% light		0.4	0.01	3.5	2.9	0.3	0.4
% moderate		0.2	N/A	1.3	1.3	0.0	0.0
% heavy		0.0	0.0	0.0	0.0	0.0	0.0
% moderate & heavy		N/A	N/A	1.3	1.3	0.0	0.0
Av. Intensity (epg):		85.5	2.0 eggs/10 mL		6010.4	67.8	108.7

**Table 3:** Prevalence of any *Schistosoma* and any STH spp in the sub-counties in the 4 Counties of Bungoma, Kakamega, Vihiga and Trans Nzoia.

Sub-County	No. of Wards	Number tested for any SCH	Number Infected by any SCH	Number tested for any STH	Number Infected by any STH	Any SCH Prevalence, (95% CI)	Any STH Prevalence, (95% CI)
***Bungoma County***							
Bumula	7	2100	242	2093	159	11.5 (10.2-13.0)	7.6 (6.5–8.8)
Cheptais	4	1199	2	1199	235	0.17 (0.04-0.7)	19.6 (17.4–21.9)
Kabuchai	4	1200	12	1199	56	1.0 (0.6-1.8)	4.7 (3.6–6.0)
Kanduyi	8	2397	151	2385	126	6.3 (5.4-7.3)	5.3 (4.4–6.3)
Kimilili	4	1204	54	1204	76	4.5 (3.4-5.8)	6.3 (5.1–7.8)
Mt. Elgon	2	596	0	589	51	0	8.7 (6.7–11.2)
Sirisia	3	900	4	900	27	0.4 (0.1-1.2)	3.0 (2.1–4.3)
Tongaren	6	1800	32	1788	45	1.8 (1.3-2.5)	2.5 (1.9–3.4)
Webuye East	3	895	90	893	64	10.1 (8.2-12.2)	7.2 ((5.6–9.1)
Webuye West	4	1200	32	1200	88	2.7 (1.9-3.7)	7.3 (6.0–9.0)
Total	45	13491	619	13450	927	4.6 (4.2-5.0)	6.9 (6.4–7.3)
***Kakamega County***							
Butere	5	1497	7	1472	107	0.5 (0.2-1.0)	7.3 (6.0–8.7)
Ikilomani	4	1185	2	1091	152	0.2 (0.04-0.7)	13.9 (12.0–16.1)
Khwisero	4	1202	6	1201	145	0.5 (0.2-1.2)	12.1 (10.3–14.0)
Likuyani	5	1502	10	1495	17	0.7 (0.4-1.2)	1.1 (0.7–1.8)
Lugari	6	1786	8	1756	31	0.5 (0.2-0.9)	1.8 (1.2–2.5)
Lurambi	6	1777	49	1697	140	2.8 (2.1-3.6)	8.3 (7.0–9.7)
Malava	7	2078	34	2047	99	1.6 (1.2-2.3)	4.8 (4.0–5.9)
Matungu	5	1503	264	1501	157	17.6 (15.7-19.6)	10.5 (9.0–12.1)
Mumias Central	4	1200	76	1200	109	6.3 (5.1-7.9)	9.1 (7.6–10.8)
Mumias East	3	900	150	900	80	16.7 (14.4-19.3)	8.9 (7.2–10.9)
Navakholo	5	1499	156	1499	116	10.4 (9.0-12.1)	7.7 (6.5–9.2)
Shinyalu	6	1799	6	1798	248	0.3 (0.1-0.7)	13.8 (12.3–15.5)
Total	60	17928	768	17657	1401	4.3 (4.0-4.5)	7.9 (7.5–8.3)
***Vihiga County***							
Emuhaya	3	900	10	900	87	1.1 (0.6-2.1)	9.7 (7.9–11.8)
Hamisi	7	2098	10	2083	174	0.5 (0.3-0.9)	8.4 (7.2–9.6)
Luanda	5	1501	14	1500	276	0.9 (0.6-1.6)	18.4 (16.5–20.4)
Sabatia	6	1801	6	1741	170	0.3 (0.1-0.7)	9.8 (8.5–11.3)
Vihiga	4	1189	4	1187	89	0.3 (0.1-0.9)	7.5 (6.1–9.1)
Total	25	7489	44	7422	796	0.6 (0.4-0.8)	10.7 (10.1–11.4)
***Trans Nzoia County***							
Cherangany	7	2093	5	2091	89	0.2 (0.1-0.6)	4.3 (3.5–5.2)
Endebess	3	900	5	900	46	0.6 (0.2-1.3)	5.1 (3.8–6.8)
Kiminini	4	1805	7	1802	70	0.4 (0.2-0.8)	3.9 (3.1–4.9)
Kwanza	6	1260	16	1260	57	1.3 (0.8-2.1)	4.5 (3.5–5.8)
Saboti	5	1498	7	1496	103	0.5 (0.2-1.0)	6.9 (5.7–8.3)
Total	25	7556	40	7549	365	0.5 (0.4-0.7)	4.8 (4.4–5.3)

In the 101 Wards where *S. mansoni* infected children were found, the proportion positive ranged from 0.1% to 33.8%, 40 Wards had *S. mansoni* prevalence ≥2%, whereas 8 Wards had heavy intensity (HI) *S. mansoni* infections ≥1%. In the 14 Wards where *S. haematobium* infected children were found, the proportion positive ranged from 0.3% to 2.7%. Only 1 Ward (Bukembe West in Bungoma county) had prevalence of *S. haematobium* ≥2%. None of the Wards had heavy intensity (HI) *S. haematobium* infections ≥1%.

### Prevalence of Soil-transmitted helminths across counties, sub-counties and wards

**Fig 5:**
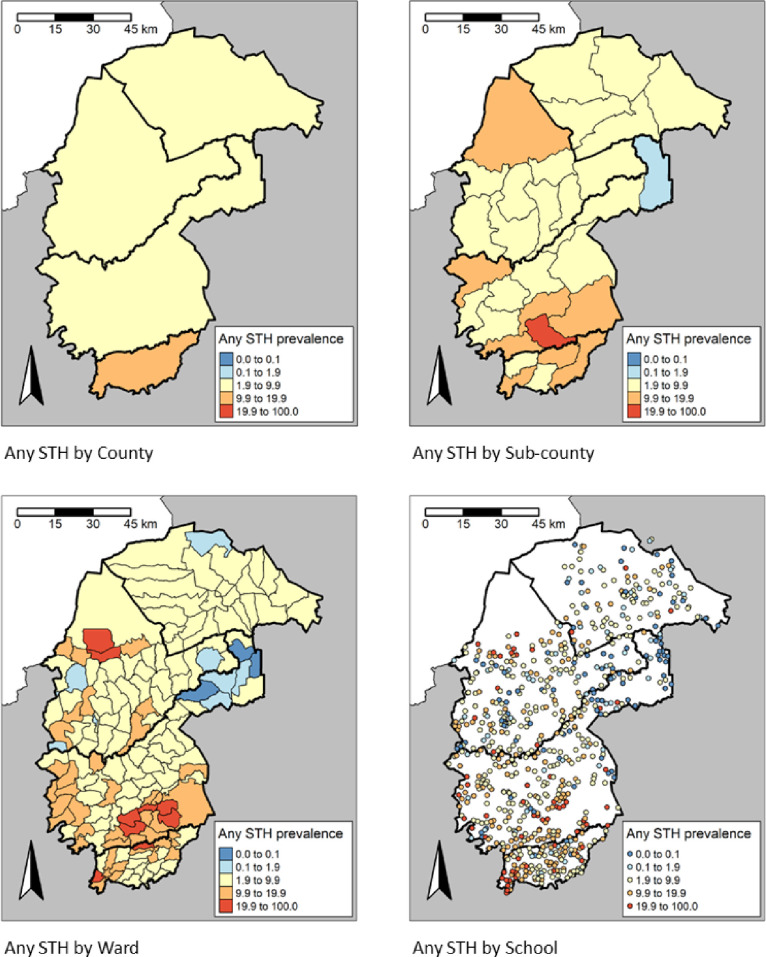
Prevalence distribution of Any STH by County, sub-County, Ward and site level, https://www.gadm.org/download_country.html.

**Fig 6:**
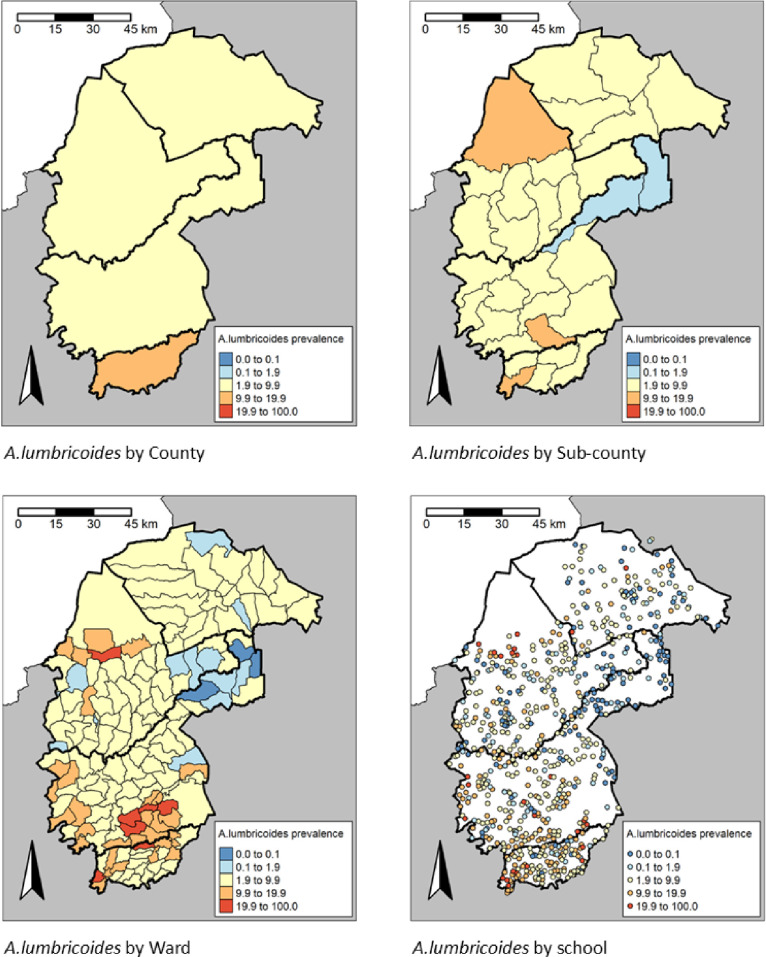
Prevalence distribution of *A. lumbricoides* by County, sub-County, Ward and site level, https://www.gadm.org/download_country.html.

**Fig 7:**
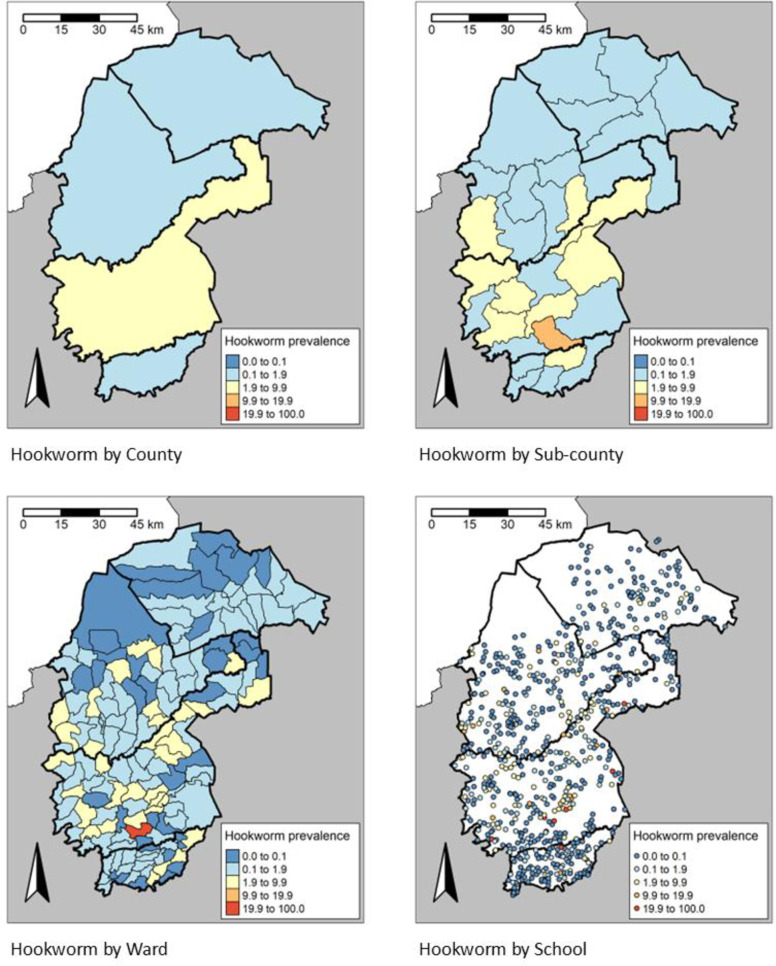
Prevalence distribution of Hookworm by County, sub-County, Ward and site level, https://www.gadm.org/download_country.html.

**Fig 8:**
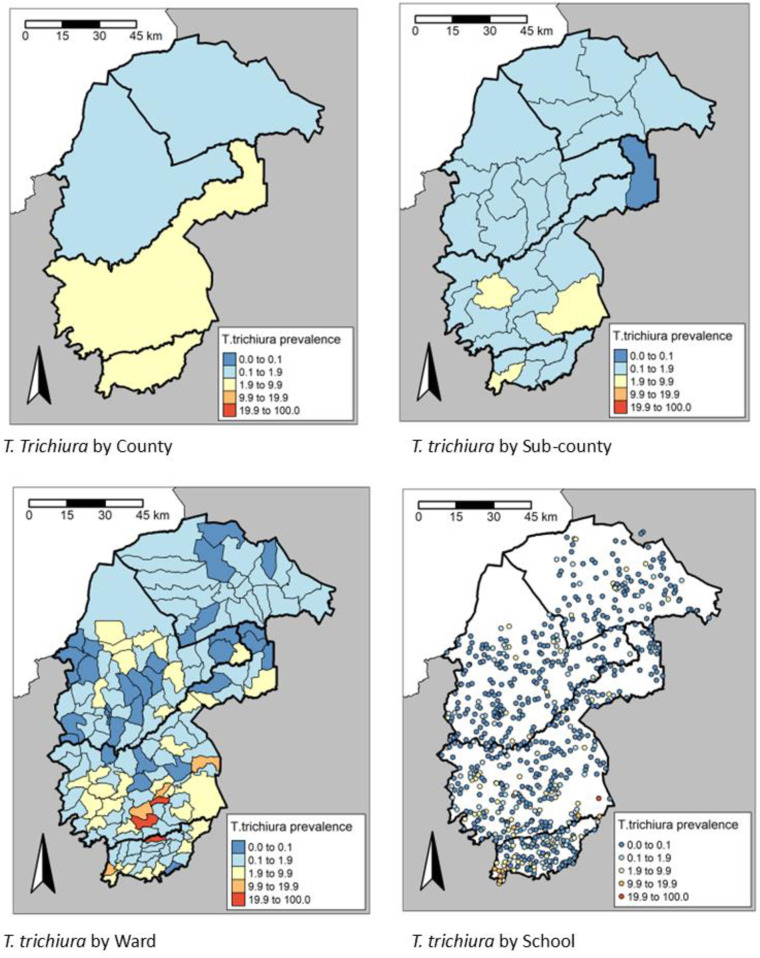
Prevalence distribution of *T. trichiura* by County, sub-County, Ward and site level, https://www.gadm.org/download_country.html.

There was a significant difference (P < 0.001) in the STH prevalence among the counties. The highest prevalence of any STH infections was observed in Vihiga county, 10.7% (95% CI: 10.1-11.5), while the lowest prevalence was in Trans Nzoia County, 4.8% (95% CI: 4.4-5.3) (**Table 2**). Cheptais sub-County in Bungoma county had the highest prevalence of any STH infections, 19.6% (95% CI: 17.4-21.9), followed closely by Luanda sub-County in Vihiga county, 18.4% (95% CI: 16.5-20.4), whereas Likuyani sub-County in Kakamega county had the lowest prevalence of any STH infections, 1.1% (0.7-1.8) (**Table 2**). The spatial heterogeneity in the prevalence of any STH across the different administrative units is presented in **Fig 5** and spatial heterogeneity in prevalence by STH species in **Figs 6**–**8**. In the 658 schools where children were found infected with any STH, the proportion positive ranged from 1.6% to 48.3%, 1.6 to 48.3% in the 636 schools with *A. lumbricoides*, 1.6% to 25.0% in the 169 schools with hookworm and 1.6% to 23.3% in the 210 schools with *T. trichiura*.

In the 152 Wards where children were found to be infected with any STH, the proportion positive ranged from 0.7% to 31.3%. 138 Wards had any STH prevalence ≥2%, whereas 59 Wards had moderate and heavy intensity (M&HI) any STH infections ≥2%. 132, 16 and 20 Wards had a prevalence ≥2% for *A. lumbricoides,* hookworm and *T. trichiura*, respectively. (Fig 7).

### Number of Wards that have achieved EPHP for SCH and STH

A summary of the number of Wards that have achieved elimination as a public health problem (EPHP), currently defined as <1% proportion of heavy intensity schistosomiasis infections and <2% proportion of STH infections of moderate and heavy intensity due to *A. lumbricoides*, hookworm and *T. trichiura* is provided in (**Table 4**). Based on the data, more than half of the Wards have already achieved the WHO target of EPHP for both SCH and STH. Specifically, 60% of Wards in both Kakamega and Bungoma counties, 44% of Wards in Vihiga county and 68% of Wards in Trans Nzoia county have attained EPHP for both SCH and STH (**Table 4**).

**Table 4:** Number of Wards that have achieved elimination as a public health problem (EPHP) for schistosomiasis and soil-transmitted helminthiasis in the 4 counties of western Kenya.

County	Total No. of Wards, n	No. of Wards achieved EPHP for SCH, n (%)	No. of Wards achieved EPHP for STH, n (%)	No. of Wards achieved EPHP for both SCH and STH, n (%)
Bungoma	45	42 (93)	29 (64)	27 (60)
Kakamega	60	55 (92)	39 (65)	36 (60)
Vihiga	25	25 (100)	11 (44)	11 (44)
Trans Nzoia	25	25 (100)	17 (68)	17 (68)

### Treatment decisions based on granular mapping data

Based on sub-County-level prevalence data, 10 sub-counties (5 each in Bungoma and Kakamega Counties) with any SCH prevalence ≥2% (**Table 3**) would require MDA with PZQ translating to 1,527,993 of the eligible population (SAC and adults). This would represent a total of 49 Wards (26 and 23 in Bungoma and Kakamega counties, respectively). Based on sub-county prevalence data, 30 sub-counties (All 10 in Bungoma, 10 out of 12 in Kakamega, all 5 in Vihiga and all 5 in Trans Nzoia county) with any STH prevalence ≥2% (**Table 3**) would require MDA with ABZ/MBZ, translating to 4,878,244 of the eligible population (PSAC, SAC and adults). This would represent a total of 144 Wards (45, 49, 25 and 25 Wards in Bungoma, Kakamega counties, respectively).

On the other hand, based on Ward-level prevalence data and ≥2% threshold for MDA, 40 Wards required MDA for SCH and 138 Wards (89.0%) required MDA for STH. However, in order to maximize on the treatment, the MoH NTD program also decided that all Wards earmarked for treatment for SCH would also receive treatment for STH (including 4 Wards that were not necessarily eligible for STH treatment), resulting in MDA for STH in a total of 142 as opposed to 138 Wards. Of the 4o Wards that met the 2% MDA threshold for SCH, 21 were in Kakamega, 17 in Bungoma, 1 in Vihiga and 1 in Trans Nzoia county. Of the 138 Wards that met the 2% MDA threshold for STH, 52 were in Kakamega, 39 in Bungoma, 25 in Vihiga and 22 in Trans Nzoia county. For the additional 4 Wards that received MDA for STH as a result of the Wards being eligible for PZQ MDA for SCH, 1 was in Kakamega (Kongoni Ward), 2 were in Bungoma (Khasoko and Milima Ward) and 1 in Trans Nzoia county (Keiyo Ward).

As a result, a total of 1,307,190 people from 40 Wards received PZQ during the CWT in December 2021 that was based on Ward-level prevalence data. Using sub-County level SCH prevalence to make treatment decisions would have resulted in overtreatment (unnecessary treatment) of 9 Wards. Using Ward-level SCH prevalence therefore ensured that overtreatment of approximately 220,803 people (representing a 14.5% reduction) was avoided, saving on approximately 715,209 PZQ tablets (representing a 16.4% reduction) (**Table 5**). On the other hand, 4,838,784 people from 138 Wards received MBZ. Using sub-County level STH prevalence to make treatment decisions would have resulted in overtreatment of 6 Wards. Using Ward-level STH prevalence therefore ensured that overtreatment of approximately 39,460 people (representing a 0.8% reduction) was avoided, saving on approximately 39,460 MBZ tablets (representing a 0.8% reduction) (**Table 5**).

**Table 5:** Comparison of the population eligible for treatment for SCH and STH based on sub-County prevalence relative to the actual treated population based on Ward-level prevalence in 2021 in the 4 counties of western Kenya.

Schistosomiasis (SCH)							
# of sub-Counties with any SCH ≥ 2%	# of Wards with any SCH ≥ 2%	Eligible population in 2021 based on sub-County prevalence (SAC: n = 461,545 Adults: n = 1,066,448)^1^	Eligible population treated in 2021 based on Ward prevalence (SAC: n = 567,145 Adults: n = 740,045)	Estimated # PZQ for eligible population based on sub-County prevalence (using x2.5 tablets for SAC and x3 tablets for adults)	Estimated # PZQ for eligible population based on Ward prevalence (using x2.5 for SAC and x3 tablets for adults)	Difference in eligible population between sub-County and Ward approaches	Difference in estimated PZQ tablets between sub-County and Ward approaches
10^2^	40	1,527,993	1,307,190	4,353,207 tablets	3,637,998 tablets	220,803 (14.5%)	715,209 tablets (16.4%)
Soil-transmitted helminthiasis (STH)							
**# of sub-Counties with any STH ≥ 2%**	**# of Wards with any STH ≥ 2%**	**Eligible population based on sub-County prevalence** (PSAC: n = 514,288 SAC: n = 1,318,174 Adults: n = 3,045,782)^3^	**Eligible population treated in 2021 based on Ward prevalence** (PSAC: n = 665,185 SAC: n = 1,666,408 Adults: n = 2,507,191)	**Estimated # ABZ/MBZ for eligible population based on sub-County prevalence** (using x1 tablet)	**Estimated # ABZ/MBZ for eligible population based on Ward prevalence** (using x1 tablet)	**Difference in eligible population between sub-County and Ward approaches**	**Difference in estimated ABZ/MBZ tablets between sub-County and Ward approaches**
30^4^	138^5^	4,878,244	4,838,784	4,878,244 tablets	4,838,784 tablets	39,460 (0.8%)	39,460 tablets (0.8%)

^1^Population estimate based on KNBS 2019 census, projected to 2021 at an inter-censal growth rate of 2.26% (Note: KNBS 2019 Census report provides this as 2.2%, but MoH DVBNTD recommended use of an average of 2.26%).

^2^Sub-Counties with SCH prevalence ≥2%: Bumula, Kanduyi, Kimilili, Webuye East, Webuye West, Lurambi, Matungu, Mumias Central, Mumias East, Navakholo.

^3^Proportions of PSAC (1–4 years), SAC (5–15 years) and Adult (15 + years) populations calculated at 10.3%, 26.4% and 61%, respectively, of the total population based on KNBS 2019 census, projected to 2021

^4^All Sub-Counties had prevalence of any STH ≥ 2% except for Likuyani and Lugari sub-Counties in Kakamega county.

^5^Excludes 4 Wards (Kongoni in Kakamega County whose prevalence of any STH was 0%; Khasoko and Milima Wards in Bungoma county, and Keiyo Ward in Trans Nzoia county whose prevalence of any STH was 1.7%), but which were included in the Community Wide Treatment of 2^nd^- 6^th^ December 2021 by the MoH NTD program because they had qualified to receive PZQ for SCH.

## Discussion

Results of the precision/granular mapping highlight the burden of schistosomiasis (SCH) and soil-transmitted helminthiasis (STH) and outlines their distribution in the 4 counties of western Kenya, including where mapping gaps existed. While the overall prevalence of any schistosomiasis in the region was 3.2%, it is noteworthy that the highest Ward level prevalence was 33.8% for Bunyala West Ward in Kakamega county. For a long time, it had been presumed that these four counties of western Kenya had either very low infection to warrant any large-scale public health intervention or were not endemic for schistosomiasis. As a result of this, these counties were systematically excluded from routine MDA for schistosomiasis. Most recent results on the impact of the NSBDP on SCH and STH documented prevalence of *S. mansoni* of 1.0%, 0.1% and 0.4% for Kakamega, Bungoma and Vihiga counties, respectively [9]. Additionally, the reported prevalences of *S. mansoni* were lower for Kakamega and Bungoma but similar for Vihiga to those observed in the current study. In the NSBDP Monitoring and Evaluation report, no surveys were undertaken for *S. haematobium*, highlighting a gap in knowledge on this species in this setting. Another study revealed *S. mansoni* prevalence of 2.2% in Bungoma County [24], whereas high snail species composition and diversity was documented in Trans Nzoia County [25] where their potential as vectors for trematodes with a possible influence on the epidemiology of SCH was suggested.

The overall prevalence of STH was 7.6%, with *A. lumbricoides* being the most common of the STH species (6.5%), and hookworm the least common (0.7%), similar to previous findings by the NSBDP [9]. The highest prevalence of any STH was in Mwibona Ward (31.3%) in Vihiga county. Previous surveys have shown a preponderance of STH in western Kenya [5] A study conducted in 3 areas (Kakamega East, South and Central) in Kakamega county reported STH prevalence of 44% [17]. The NSBDP survey conducted between 2012–2017 documented STH prevalence of 9.8%, 7.3% and 32.8% for Kakamega, Bungoma and Vihiga Counties, respectively [9]. Relative to the NSBDP survey (which excluded Trans Nzoia county), the prevalence of any STH reduced slightly for Kakamega (9.8% vs 7.9%) and Bungoma (7.3% vs 6.9%) counties, and reduced substantially for Vihiga county (32.8% vs 10.7%) in the present survey. Earlier studies reported much higher prevalences of STH in this region; 26.8% for Kakamega County [18] and 14.2% [15], 27.6% [16] and 24.7% [24] for Bungoma County. The low STH prevalence noted in the present study is not surprising and may be attributable to deworming targeting STH in these areas over the years by the NSBDP [10].

The spatial distribution of SCH and STH in western Kenya is influenced by ecological factors such as climate, water sources, and agricultural practices [2,4,5]. Higher SCH prevalence in Bungoma (4.6%) and Kakamega (4.3%) may be linked to wetter conditions that support snail populations, whereas lower prevalence in Trans Nzoia (0.5%) may reflect drier conditions. Proximity to freshwater bodies, including the Yala and Nzoia rivers, plays a key role in SCH transmission, particularly through water-contact activities such as fishing and laundry. Agricultural activities, particularly irrigation and livestock farming, contribute to both SCH and STH transmission by increasing water exposure and soil contamination. However, potential biases in site selection, which prioritized high-risk areas near water sources, may have influenced prevalence estimates for SCH. Future studies should integrate spatial modeling of environmental variables, randomized site selection, and snail habitat surveys to refine risk assessments and intervention strategies.

Based on the data from the current survey, more than half of the Wards have already achieved the WHO target of EPHP for both SCH and STH, presenting good prospects for pushing forward with interruption of transmission (IoT) as envisaged in the BTS in these counties. Complementary interventions such as water, sanitation and hygiene (WASH) and behaviour change and communication (BCC) as envisaged in the BTS will go a long way in not only sustaining the chemotherapeutic gains accrued from the years of MDA by the NSBDP, but also in accelerating attainment of IoT of SCH and STH in these four counties. If the Ward-level SCH and STH prevalences remain below 2%, and the prevalences for HI (for SCH) and M&HI (for STH) remain below 1% and 2%, respectively, after the mid-term survey (after 2 rounds of MDA), the program may consider switching from MDA to other innovative treatment approaches in Wards that qualify. These approaches include test and treat strategies for instance *test-treat-track-test-treat* (T5) for SCH [26], active surveillance and other passive surveillance approaches for individuals who present at health facilities, ensuring medicines are available to treat individual suspected cases at peripheral health facilities among other cost-effective and sustainable methods. Such approaches would not only address the BTS but would also be in consonance with WHO’s guidelines that discourage widespread treatment in areas of very low SCH (<10%) [23] and very low STH prevalence (<20%) (WHO, 2019). The recommendation to use both sustained low prevalence (<2%) and EPHP cut-offs in making the switch from MDA to other innovative approaches takes into consideration recent concerns that, for SCH at least, that the current definition of EPHP is inadequate and outdated, and the shifting distribution of SCH morbidity towards more subtle, rather than severe, morbidity in the face of large-scale control programmes requires guidelines to be adapted [27]. In this regard, there are increasing calls on the need to use overall prevalence of SCH (instead of the prevalence of heavy intensity infections) in the M&E framework for SCH in order to achieve the goal of widespread EPHP of SCH and to meet the WHO road map targets [27–30].

The prevalence and distribution of SCH and STH infections in the present survey had several implications for MDA. Whereas the WHO recommends MDA for SCH and for STH wherever the prevalence of infection exceeds 10% and 20%, respectively, [23,31], the Kenya NTD program through the BTS [13] has set forth a 2% threshold for implementation of MDA, with the Ward level as the implementation unit. The goal of BTS is breaking transmission in areas where interruption of transmission is feasible rather than elimination of the diseases as a public health problem. This was based on the appreciation that aggregation of SCH and STH data at higher administrative levels such as County and sub-County hides considerable spatial heterogeneity. Indeed, in the present study, the selection of 5 schools (sites) per Ward for sampling achieved a ~ 5-fold finer resolution compared to if the 5 schools were to be selected from an entire sub-County. Consequently, this shift from sub-County to Ward-level implementation strategy impacted treatment decisions. Use of Ward prevalence for SCH resulted in significant reduction in the number of people eligible for treatment by 14.5% with a concomitant reduction of over 16% in the number of PZQ tablets. These findings align with WHO’s NTD Roadmap 2021–2030 in terms of improving the accuracy of MDA targeting, reducing overtreatment and supporting the transition from morbidity control to interruption of transmission (IoT). WHO advocates for data disaggregation at the IUs, to the sub-district or Ward level during planning and implementation of MDA to allow for precise estimation of risk, better targeting, and efficient resource allocation [32]. The Ward-level approach used in Kenya demonstrates the value of micro-mapping, which could inform WHO’s refinement of mapping strategies to move beyond district-level prevalence estimates.

On the other hand, the reduction in the number of persons in need of treatment for STH (0.8%) although not to the same scale as for SCH, would be substantial if larger endemic areas and/or an entire 5-year MDA cycle (cumulative effects) are considered. This modest reduction also reflects the largely homogenous distribution of STH unlike SCH. By avoiding overtreatment (unnecessary treatment), the Ward-level implementation therefore revealed the hidden benefits of precision mapping and allowed for optimal targeting of treatment to those that really needed it. Furthermore, it provided an opportunity for the reallocation of the saved resources to other endemic areas in need. Such savings in resources are expected to improve moving forward, especially in light of better/enhanced survey protocols such as the Practical and Precision Assessment that has recently been added to the WHO survey armamentarium of possible approaches to conducting surveys for SCH/STH epidemiological assessments.

Data from the precision mapping survey has enabled the Kenya National NTD control program to accurately forecast medicines needed for treatment, and for domestic resource mobilization. In addition, the survey has also allowed sub-Counties in the SCH endemic areas to include PZQ in their list of essential drugs requests to the central drugs store - Kenya Medical Supplies Agency.
